# Surgery

**Published:** 1897-12

**Authors:** James Swain


### SURGERY.

In an address delivered before the Congress of American Physicians and Surgeons, and also at the Twelfth International Congress recently held in Moscow, Dr. N. Senn[2] treated of the classification of **acute peritonitis**, which, with its varieties and classes, makes a grand total of something like sixty different forms. This result is suggestive that either the surgery of the peritoneum is in a very advanced or a very chaotic condition.

In truth, however, it is in neither state; for while, on the one hand, we no longer regard a wound of the peritoneum as more dangerous than a wound of any other important structure, yet we have much to learn with reference to the causes and treatment of inflammations of the peritoneum, and particularly of that form known as **general purulent peritonitis** which is commonly associated with some perforative lesion of the abdominal viscera. Under this heading, however, different forms of disease have been classed, for whereas Quénu, Bouilly, Chaput,[3] and many others speak of recovery from such cases as not particularly uncommon, Weir[4] does not stand alone in his experience of twenty cases without a single operative success. Senn is strongly of opinion that recoveries from so-called general peritonitis were cases operated upon before the entire serous surface was involved, or where there was a real, though not apparent, limitation of the inflammation. He rightly draws a distinction between general septic peritonitis—in which the patients die so soon after the beginning of the disease that at the necropsy or, if the abdomen is opened during life, at the operation no gross

[2] *Am. J. M. Sc.*, 1897, cxiv. 136; *Med. Rec.*, 1897, lii. 289.

[3] *Med. Week*, 1897, v. 250.

[4] *Ann. Surg.*, 1897, xxvi. 235.

tissue change is found—and acute suppurative peritonitis which results in the formation of pus and is always more or less circumscribed. Although we may regard the former as essentially a streptococcal infection, and the latter as a staphylococcal infection, there is still much room for working out the bacteriology of this class of inflammation, and from this we may expect a more perfect understanding of the differences of the two forms. Most observers agree that the more lymph found in the peritoneal cavity the better the chance of recovery, but that under any circumstances the so-called general suppurative peritonitis is an extremely fatal form of disease and one in which most of us have been disappointed in our operative results.

McCosh,[1] who is a believer in a **general suppurative peritonitis** unlimited by adhesions, appears, by a more radical procedure than that usually employed, to have saved six cases out of forty-three upon which he has operated—a mortality of about eighty-six per cent. His method is founded on a belief that by cathartics we can best promote peristalsis in the intestinal paralysis which occurs in this condition, and we thus increase intestinal drainage and diminish the danger of the emigration of the bacillus coli communis from the intestinal walls. The plan of treatment which he employs is as follows: Chloroform is administered and an incision five or six inches long is made in the abdominal wall over the organ which has excited the peritonitis, and the patient is turned on the side so as to favour the escape of the purulent fluid. Unless there is a great deal of distension or the heart's action is very weak, the intestines are allowed to escape from the abdominal cavity into hot towels held by an assistant. Any cause of the peritonitis which is found is then treated, such as the removal of a gangrenous appendix or suturing perforated intestine. The cavity of the peritoneum and the intestines are then thoroughly irrigated with normal salt solution. Gallons of fluid at a temperature of 110° to 112° F. are used, and every nook and corner must be opened up by the hands of the operator so that they may be washed with the solution. A considerable amount of this fluid is left in the abdominal cavity for the purpose of stimulating the heart and of favouring intestinal drainage. If there is any difficulty in returning the bowels they must be incised so as to allow flatus to escape, and the incisions are then closed with Lembert sutures. One or two ounces of a saturated solution of sulphate of magnesium are then injected through a hollow needle attached to an aspirating syringe as high up in the ileum or jejunum as can be conveniently reached, and the needle puncture is closed by a Lembert suture. The peritoneal cavity is generally drained by four or more strips of sterile gauze thrust in different directions among the intestines, and supplemented if necessary by a large glass tube inserted into the

[1] *Ann. Surg.*, 1897, xxv. 687.

pelvis. The abdominal wound is only partially closed, so as to afford freer exit for the escape of peritoneal secretions. The edges are not closely approximated but only partially drawn together by two or three silkworm-gut sutures, between which and the intestines is placed a gauze compress. A ten-grain dose of calomel is given on the return of the patient to bed, and rectal stimulation is employed for the first twenty-four or thirty-six hours. The great point in favour of this intra-intestinal injection of cathartics is, that the vomiting which occurs in this disease prevents them being given by the mouth and when administered by the rectum they are unsatisfactory. Senn[1] speaks well of their use and further advises the subcutaneous injection of Marmorek's antistreptococcic serum, but Dr. Weir[2] has not had favourable results in the cases in which he has tried sulphate of magnesium injected into the intestines or subcutaneously.

Dr. Finney[3] also comes forward with a method which he claims has been instrumental in five recoveries. It is based on the well-known power of the peritoneum to absorb and dispose of a relatively large amount of infectious material—a function which he believes to be greatly impaired by the large amount of exudate covering the peritoneum in cases of general suppurative peritonitis. The plan of treatment which he advocates is as follows: An incision long enough to allow of free access to all parts of the abdominal cavity is made over that part from which the peritonitis is supposed to spring. The coils of intestine are quickly drawn out into warm towels, beginning with those which are most affected, and the peritoneal cavity is then thoroughly wiped with pledgets of gauze soaked in normal salt solution—especial care being given to the pelvis. Dr. Finney thinks it rarely necessary to flush the cavity with salt solution. Then the intestines which are lying outside the abdominal cavity are similarly wiped with pledgets of gauze from one end to the other, in order to remove the adherent flakes of partially organised lymph. This cleaning process sometimes requires the use of considerable force, and is facilitated by being carried on under a constant stream of warm salt solution. The intestines are then returned, the most damaged portion being returned last so that it may be most superficial and better drained by gauze which is packed around it if necessary. The abdominal wound is then closed, and the calomel followed by salts administered by the mouth and aided by a turpentine enema.

Weir[4] and Abbe[5] are opposed to this removal of lymph and regard its presence as protective. The raw surface which is left is probably also more favourable for infection. This is the view the majority of surgeons would take; but the obvious

[1] *Loc. cit.* [2] *Loc. cit.*
[3] *Johns Hopkins Hosp. Bull.*, 1897, viii. 141; editorial in *N. York M. J.*, 1897, lxvi. 226.
[4] *Loc. cit.* [5] *Ann. Surg.*, 1897, xxvi. 236.

lesson to be learnt from these two methods—which after all have a great deal in common in their fundamental principles—is, that by a more careful and thorough cleansing of the peritoneum than it has hitherto been considered justifiable to adopt we may hope to diminish the terrible mortality in this fatal condition.

In some of the cases above refered to the abdomen was closed without **drainage of the peritoneal cavity.** Drainage is adopted much less frequently now than formerly; but Dr. Clark,[1] of Baltimore, maintains that it is still too frequently employed, and that with more attention to details of operation and greater reliance on the ability of the peritoneum to get rid of infectious material we may overcome many difficulties which are now incorrectly supposed to be obviated by drainage. The immense power of absorption of the peritoneum was well shown by Wegner's experiments on dogs; and according to this observer the surface of the peritoneum is about 17,182 square centimetres—nearly equivalent to that of the skin—and in one hour is able to absorb three to eight per cent. of the body weight. Unfortunately, however, it is capable under irritation of allowing the transudation of an equal amount into the peritoneal cavity. From a study of the writings of Grawitz,[2] Cobbett and Melsome[3] and others, Clark concludes that: 1. Under normal conditions the peritoneum can dispose of pyogenic organisms in varying quantities, depending upon the virulence of the organism, without producing peritonitis. 2. The less the absorption from the peritoneal cavity, the greater the danger of infection. 3. Solid sterile particles are partly absorbed, and the remainder is encapsuled without the production of peritonitis. 4. Death may be produced by general septicæmia, and not peritonitis, where large quantities of organisms are taken up by the lymph streams. 5. Peritonitis may be produced if the culture fluid is difficult of absorption. Irritant material which destroys the tissues of the peritoneum prepares a place for the lodgment of organisms and the starting place for peritonitis. 7. An infected stitch-hole tract or a localised phlegmon communicating with the peritoneum forms an excellent starting place for general peritonitis. 8. Stagnation of degenerated fluid in "dead" spaces favours the growth of organisms. 9. The presence of infected blood-clots is especially liable to cause a virulent peritonitis. 10. Injury to the abdominal viscera, such as strangulation of an intestine, constriction and ligation of large areas of tissue in the presence of pyogenic organisms, will almost certainly be followed by peritonitis.

Arguing from these experimental data, Clark considers that attention to the following details of operation are most useful in avoiding the supposed necessity for drainage: The hands must

1 *Am. J. Obst.*, 1897, xxxv. 481, 650.
2 *Charité-Ann.*, 1886, xi. 770; cited by Clark.
3 *J. Path. & Bacteriol.*, 1896, iii. 39.

be most carefully disinfected, and it is safer to suspend all operations for at least three days after contact with an infectious case, as suggested by Zweifel. All hemorrhage must be carefully stopped; but even if oozing continues, it is safer to leave the peritoneum to take care of it than to drain. As little as possible of the peritoneum must be sacrificed, and bruising or otherwise injuring the tissues must be avoided. The general peritoneal cavity should be isolated as much as possible during operation by means of gauze pads. The intestines must never be allowed to become chilled by exposure, for Wegner has shown that a lowering of temperature diminishes resistance to infection and increases the transudation of serum without compensatory absorption. Great care should be taken to avoid rupture of an abscess in the peritoneal cavity, and after every operation where *débris* or normal fluids have escaped the abdomen should be thoroughly irrigated with normal saline solution. Absorption is promoted by the introduction of 500 to 1000 cubic centimetres of salt solution into the peritoneal cavity if the presence of septic matter is suspected, and is also aided by elevating the foot of the bed for the first twenty-four hours after operation. This last suggestion is founded on Muscatello's[1] experiments, which show that the peritoneum covering the diaphragm has the greatest power of absorption, and, moreover, it drains any fluid which has collected in the pouch of Douglas or other cavity. As this is a prophylactic and not a curative measure, it should not be employed in cases where peritonitis is already established. Should symptoms of infection arise after operation, Clark is in favour of the injection of about a litre of saline solution into the cellular tissue under the breasts, as recommended by Bosc and Claisse. He admits, however, that in certain cases drainage is necessary: (1) In appendicitis when the peritoneum and tissues adjacent to the appendix are infiltrated with inflammatory products, preventing a secure closure of the stump after amputation of the appendix, and when the appendix has ruptured and either caused a localised abscess or a general peritonitis. In early cases drainage is not required, and a distinct objection to its use, here and elsewhere, is the liability of post-operative hernia occurring in its track. (2) Localised collections of pus in the pelvis.—In these cases either the abscess sac should be cleanly enucleated and the abdomen closed without drainage, or it should not be opened through the abdomen if it is too adherent to be enucleated with safety. These cases are, *par excellence*, the ones for incision and drainage through the vagina, as shown by sixty cases thus treated in the Johns Hopkins Hospital with only two deaths. (3) In suture of the intestine a drain should be employed when there is doubt as to the integrity of the suturing. (4) Excision of fistulous tracts leading from the intestine to the abdominal wall.—In these cases it is safer to pack a gauze drain down to the sutured

[1] *Arch. f. path. Anat.* [etc.], 1895, cxlii. 327.

areas in the intestine, for they are especially prone to break down and re-establish the track. (5) In purulent peritonitis Pawlowsky has shown that the usual avenues for the absorption of fluids from the abdominal cavity are closed, consequently we can only endeavour to supplement them by thorough irrigation of the abdominal cavity and free drainage. Finney,[1] however, has shown in the cases referred to above that even in purulent peritonitis drainage may be dispensed with.

As Warbasse[2] says in criticising Clark's paper, the dangers of drainage have probably been overestimated, although it is always advisable to completely close the abdomen whenever possible. It is during the first few hours when drainage is needed. That is the time when irritated areas are becoming walled off by their own natural efforts, and when it is most important that the products of these areas should be conducted away. The drain having served its purpose, its further usefulness consists in draining the cavity which it has made for itself; and in the treatment of this cavity the surgeon need anticipate no difficulty.

JAMES SWAIN.

### LARYNGOLOGY AND RHINOLOGY.

[1] *Loc. cit.* [2] *Ann. Surg.*, 1897, xxvi. 250.

